# The Hormonal–Metabolic Puzzle of PCOS: Linking AMH Levels, Body Fat Distribution, and Insulin Resistance in Overweight and Obese Women

**DOI:** 10.3390/metabo16050295

**Published:** 2026-04-27

**Authors:** Amalia Gorzko, Jolanta Nawrocka-Rutkowska, Agnieszka Brodowska, Edyta Śliwak, Andrzej Starczewski, Iwona Szydłowska

**Affiliations:** Department of Gynecology, Endocrinology and Gynecological Oncology, Pomeranian Medical University, ul. Rybacka 1, 71-252 Szczecin, Poland; jolanta.nawrocka.rutkowska@pum.edu.pl (J.N.-R.); agnieszka.brodowska@pum.edu.pl (A.B.); edyta.sliwak@gmail.com (E.Ś.); andrzej.starczewski@pum.edu.pl (A.S.); iwona.szydlowska@pum.edu.pl (I.S.)

**Keywords:** PCOS, AMH, insulin resistance, WHR, BMI

## Abstract

**Background**: The relationship between AMH (anti-Müllerian hormone) levels, fat distribution, and insulin resistance in women with PCOS has been widely studied, yet findings remain inconsistent. Recent guidelines emphasize the growing role of AMH in PCOS diagnosis and suggest its potential inclusion among diagnostic criteria, highlighting its relevance for guiding therapeutic management. **Objectives**: This retrospective study aimed to evaluate the association between AMH levels and metabolic parameters in overweight and obese reproductive-age women with PCOS. Ethical approval was obtained from the bioethics committee. **Methods**: Two groups of 52 women each were selected from PCOS patients treated at our clinic between 2024 and 2025: one with a waist-to-hip ratio (WHR) ≤ 0.85 and the other with a WHR > 0.85. Venous blood samples were collected to measure AMH, fasting glucose, and fasting insulin. Body height and weight were recorded to calculate body mass index (BMI), and insulin resistance was assessed using HOMA-IR. Waist and hip circumferences were measured to determine WHR. **Results**: The association between central adiposity and AMH in overweight and obese women with PCOS depended on insulin resistance. In insulin-resistant women, higher WHR was linked to lower AMH, whereas in women without insulin resistance, higher WHR corresponded to higher AMH levels. **Conclusions**: Insulin resistance appears to influence AMH in opposite directions depending on a woman’s WHR, suggesting its potential role in tailoring individualized treatment strategies.

## 1. Introduction

Polycystic ovary syndrome (PCOS) affects 10–15% of women of reproductive age and is characterized by a wide spectrum of reproductive and metabolic disturbances, including increased risk of metabolic syndrome, type 2 diabetes, and cardiovascular disease, largely driven by insulin resistance, hyperinsulinemia, dyslipidemia, and central obesity. According to the 2003 Rotterdam criteria, PCOS is diagnosed when at least two of the following are present: oligo- or anovulation, clinical or biochemical hyperandrogenism, or polycystic ovarian morphology on ultrasound [1]. Recently, anti-Müllerian hormone (AMH) has been proposed as a reliable alternative to ultrasound for assessing ovarian reserve [1]. Produced by granulosa cells of preantral and small antral follicles, AMH reflects both follicle quantity and maturation, and its levels are influenced by age, hormonal contraceptives, smoking, and ethnicity, among others [2].

Obesity, defined as an excess of body fat resulting from a prolonged positive energy balance, is a growing public health challenge [1]. While body mass index (BMI) is widely used to screen for obesity, it does not capture fat distribution or predict metabolic risk, limiting its utility in PCOS [3,4,5]. Waist circumference and waist-to-hip ratio (WHR) are more informative measures of central adiposity. WHR is calculated as waist circumference divided by hip circumference, providing a simple estimate of fat distribution; values > 0.85 indicate central (android) obesity, while values ≤ 0.85 reflect gluteofemoral (gynoid) fat accumulation [1].

Women with PCOS frequently exhibit central obesity, which is strongly associated with metabolic disturbances, hormonal dysregulation, and adverse reproductive outcomes [6,7,8]. Excess abdominal adipose tissue contributes to insulin resistance, stimulates excessive androgen production, and promotes chronic low-grade inflammation, with markers such as hsCRP (High-Sensitivity C-Reactive Protein) and proinflammatory cytokines (e.g., IL-6, IL-18) primarily produced by visceral fat [6,7]. Insulin resistance, particularly prevalent in overweight and obese women with PCOS, further aggravates metabolic and hormonal imbalances by impairing insulin signaling through inflammation and elevated free fatty acids [9,10,11].

Despite growing interest in the role of anti-Müllerian hormone (AMH) in polycystic ovary syndrome (PCOS), its relationship with indices of fat distribution remains insufficiently defined. Existing studies evaluating anthropometric parameters, particularly waist-to-hip ratio (WHR), have reported inconsistent findings, and data on the potential modifying effect of insulin resistance are limited. This indicates an important gap in understanding the interplay between central adiposity, metabolic status, and ovarian reserve in women with PCOS [1,2].

Given the high prevalence of central obesity in PCOS and its significant impact on metabolic and endocrine function, we aimed to investigate its relationship with AMH. By examining WHR alongside insulin resistance and other metabolic parameters, our study seeks to clarify the role of central adiposity in ovarian reserve, filling a gap in current research and informing potential individualized interventions.

## 2. Patients and Methods

### 2.1. Study Group

This research was designed as a retrospective study and involved participants from the Polish population aged between 18 and 39 years. Women older than 39 years were excluded to minimize the confounding effect of age-related decline in AMH and ovarian reserve. Before enrollment, all participants received written information outlining the aims and methodology of the study. Each participant provided written informed consent and agreed to the processing of personal data in accordance with Regulation (EU) 2016/679 of the European Parliament and of the Council of 27 April 2016 concerning the protection of natural persons with regard to the processing of personal data (Official Journal of the European Union L 119, 4 May 2016, p. 1). Ethical approval for the study was obtained from the Bioethics Committee of the Pomeranian Medical University (approval no. KB-006/12/2024).

The study included women diagnosed with polycystic ovary syndrome according to the Rotterdam criteria. Medical histories focusing on general health status were obtained from all participants. Women with a known history of systemic diseases, including type I or type II diabetes mellitus, thyroid dysfunction (hypothyroidism or hyperthyroidism), hypertension, cardiovascular disorders, or liver disease, were excluded, as these conditions could potentially confound the study outcomes. All hormonal medications were discontinued at least three months prior to inclusion. Gynecological examination and transvaginal ultrasonography were performed in all patients to evaluate the reproductive organs. Ovarian assessment included measurements of ovarian volume and evaluation of follicle number, diameter, and distribution. Ultrasonographic examinations were conducted using an Alpinion X-CUBE 70 ultrasound system equipped with an 11 MHz endovaginal transducer. All clinical, biochemical, and ultrasonographic variables were collected during routine diagnostic work-up using standardized clinical protocols.

Serum was obtained from each participant for the assessment of AMH, fasting glucose, and fasting insulin concentrations. Insulin resistance was evaluated using the HOMA-IR index, derived from fasting glucose and insulin measurements. A HOMA-IR threshold greater than 2.5 was used to define insulin resistance.

Patients’ weight and height were measured to calculate body mass index (BMI). BMI values were classified in accordance with World Health Organization criteria into the following categories: underweight, normal weight, overweight, and obesity.

This study was designed as a retrospective analysis of prospectively collected clinical and laboratory data. From the entire population of PCOS Polish patients (194 women) hospitalized in the Department of Gynecology, Endocrinology and Gynecological Oncology, Pomeranian Medical University in Szczecin, Poland, and treated at the affiliated outpatient clinic between 2024–2025, all individuals with a waist-to-hip ratio (WHR) ≤ 0.85 and BMI > 25 were identified. A total of 68 patients met this criterion. Subsequently, a comparison group was selected from the remaining PCOS patients presenting during the same period who had a WHR > 0.85. A 1:1 matching procedure using propensity score matching (PSM) was applied, whereby each patient in the WHR ≤ 0.85 group was matched with a corresponding patient in the WHR > 0.85 group selected from a pool of 126 eligible individuals. This procedure resulted in two equally sized study groups that were comparable with respect to age and BMI. The final analytical sample consisted of 104 women, including 52 with WHR ≤ 0.85 and 52 with WHR > 0.85.

**Figure 1:**
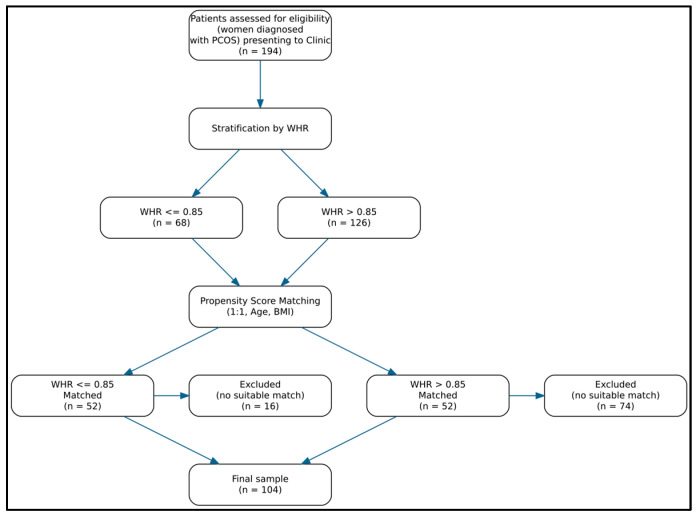
Flowchart of patient selection, stratification by WHR, and propensity score matching.

The matching strategy was applied to minimize the influence of potential confounding factors, particularly age and overall adiposity, and to allow assessment of the association between adipose tissue distribution, expressed as WHR, and anti-Müllerian hormone (AMH) levels independently of these variables. Figure 1 shows the distribution of patient selection.

### 2.2. Biochemical Tests

Serum samples (5 mL of venous blood) were collected on days 3–5 of the menstrual cycle (early follicular phase). All analyses were performed in the same laboratory. AMH was measured using the Elecsys AMH Plus electrochemiluminescence immunoassay (Roche Diagnostics, Mannheim, Germany) on a Cobas e analyzer. Glucose levels were determined using an enzymatic hexokinase method on a Cobas analyzer, while insulin concentrations were measured using an electrochemiluminescence immunoassay (Roche Diagnostics, Mannheim, Germany) on a Cobas e system.

### 2.3. Statistical Analysis

Statistical analyses were performed using R (version 4.5.0). Two-way ANOVA was conducted using base R functions from the stats package. Propensity score matching was implemented using MatchIt, with balance diagnostics performed using cobalt. Linear regression models with cluster-robust standard errors were fitted using the sandwich and lmtest packages. Data manipulation and visualization were performed using dplyr and ggplot2.

Continuous variables were assessed for normality using the Shapiro–Wilk test, supported by Kolmogorov–Smirnov and Lilliefors tests. Due to a non-normal distribution of anti-Müllerian hormone concentrations, AMH values were logarithmically transformed (natural logarithm, ln[AMH]) prior to further analyses.

Descriptive statistics are presented as mean ± standard deviation (SD) for approximately normally distributed variables. Categorical variables are presented as counts and percentages.

To ensure comparability between groups, propensity score matching was applied. The propensity score was estimated using a logistic regression model in which the probability of belonging to the WHR > 0.85 group was modeled as a function of age and BMI. Subsequently, 1:1 nearest-neighbor matching was performed with exact matching within age strata, and women older than 39 years were excluded. Matching quality was assessed using standardized mean differences (SMDs), achieving very good balance across all covariates (SMD < 0.1).

Initial comparisons of ln(AMH) between WHR categories (≤0.85 vs. >0.85) were performed using independent-samples *t* tests. A two-way analysis of variance (ANOVA) was applied with the WHR category (≤0.85 vs. >0.85) and insulin resistance status (IR+ vs. IR−) as fixed factors, including their interaction term. In the presence of a significant interaction, simple effects were examined using pairwise comparisons within levels of the moderating factor. All statistical tests were two-sided, with a significance threshold of *p* < 0.05. Where appropriate, results with *p* values between 0.05 and 0.10 were interpreted as statistical trends.

A linear regression model was fitted with log-transformed anti-Müllerian hormone concentration (lnAMH) as the dependent variable. The independent variables included waist-to-hip ratio category (WHR > 0.85 vs. WHR ≤ 0.85), insulin resistance status (IR− vs. IR+), and their interaction term (WHR × IR). Women with WHR ≤ 0.85 and insulin resistance (IR+) constituted the reference category. Because the analyses were performed on data obtained after 1:1 propensity score matching, standard errors were estimated using cluster-robust variance estimators, with clustering at the matching subclass level, in order to account for within-pair dependence.

In the residual diagnostics Shapiro–Wilk and Breusch–Pagan tests were used.

## 3. Results

The analysis was restricted to women with PCOS aged 18–39 years. Subsequently, PSM was performed using a 1:1 nearest-neighbor algorithm with a caliper of 0.10, enforcing exact matching within five-year age strata (18–20, 21–25, 26–30, 31–35, and 35–39 years). Balance between groups was assessed using standardized mean differences (SMDs), with values below 0.10 indicating good balance.

Table 1 presents baseline characteristics of the matched cohort stratified by waist-to-hip ratio (WHR ≤ 0.85 and WHR > 0.85) derived from the original cohort. Continuous variables are presented as mean ± standard deviation. Categorical variables are presented as counts and percentages. Anti-Müllerian hormone concentrations and log-transformed AMH values (lnAMH) are shown descriptively; no formal hypothesis testing of baseline characteristics was performed after matching.

The distribution of AMH significantly deviated from normality in both groups (Shapiro–Wilk test, *p* < 0.001). After logarithmic transformation, the lnAMH distribution conformed to normality. Comparison of lnAMH using Welch’s *t*-test revealed no statistically significant differences between the groups (*p* = 0.31).

A two-way analysis of variance (ANOVA) was conducted to examine the effects of waist-to-hip ratio (WHR category) and insulin resistance status on log-transformed anti-Müllerian hormone levels (lnAMH). The model included WHR group (≤0.85 vs. >0.85), insulin resistance (IR+ vs. IR−), and their interaction.

There was no significant main effect of WHR group on lnAMH (F(1,100) = 1.13, *p* = 0.29), nor a significant main effect of insulin resistance (F(1,100) = 0.46, *p* = 0.50). In contrast, a significant WHR × insulin resistance interaction was observed (F(1,100) = 10.81, *p* = 0.001), indicating that the association between WHR and AMH differed according to insulin resistance status.

Post hoc simple effects analyses demonstrated that among insulin-resistant women (IR+), lnAMH was significantly lower in those with WHR >0.85 compared with WHR ≤ 0.85 (*p* = 0.009). Conversely, among women without insulin resistance (IR−), lnAMH was significantly higher in the WHR > 0.85 group compared with WHR ≤ 0.85 (*p* = 0.041). When insulin resistance status was examined within WHR strata, lnAMH differed significantly by insulin resistance only in the WHR > 0.85 group (*p* = 0.009), whereas no significant difference was observed within the WHR ≤ 0.85 group (*p* = 0.11).

**Figure 2:**
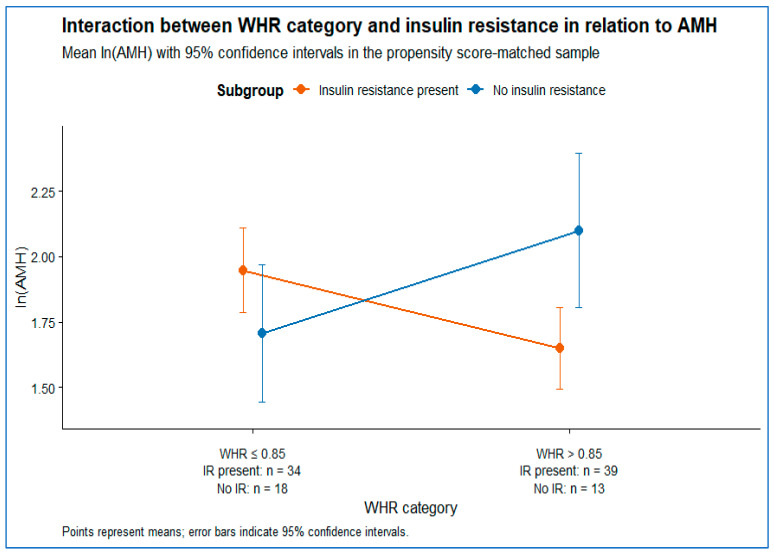
Interaction between WHR category and insulin resistance in relation to AMH.

This interaction is illustrated in Figure 2, which shows crossing mean lnAMH profiles across WHR categories by insulin resistance status.

The estimated regression equation was as follows:

lnAMH = 1.948 − 0.298 (WHR > 0.85) − 0.243 (IR−) + 0.693 (WHR > 0.85 × IR−)

The regression analysis demonstrated a statistically significant interaction between WHR category and insulin resistance status (β_3_ = 0.693, *p* < 0.001), indicating that the association between central adiposity and AMH concentration differed according to insulin resistance in women with PCOS.

In the reference group of women with WHR ≤ 0.85 and insulin resistance, the estimated mean lnAMH was 1.95. Among insulin-resistant women, a WHR greater than 0.85 was associated with a significantly lower lnAMH (β_1_ = −0.298, *p* = 0.008), corresponding to an approximate 26% reduction in AMH concentration.

In contrast, among women without insulin resistance, WHR > 0.85 was associated with higher lnAMH values as a result of the positive interaction effect. The combined effect of WHR > 0.85 in insulin-sensitive women (β_1_ + β_3_ = 0.395) corresponded to an approximately 48% higher AMH concentration compared with insulin-resistant women with WHR ≤ 0.85.

The main effect of insulin resistance alone was not statistically significant (β_2_ = −0.243, *p* = 0.104), suggesting that its association with AMH is conditional on WHR status rather than independent.

After back-transformation of the regression coefficients to the original AMH scale, WHR > 0.85 was associated with a 25–30% reduction in AMH among insulin-resistant women. In contrast, among insulin-sensitive women, WHR > 0.85 was associated with nearly a twofold increase in AMH concentration relative to insulin-resistant women with WHR ≤ 0.85. These findings may indicate that insulin resistance substantially modifies both the direction and magnitude of the association between central adiposity and AMH levels.

Residual diagnostics did not indicate meaningful violations of model assumptions. Residuals were approximately normally distributed (Shapiro–Wilk *p* = 0.47), and there was no evidence of heteroskedasticity (Breusch–Pagan *p* = 0.94). Although a small number of observations showed moderate leverage or influence, none exerted undue impact on the model estimates, supporting the robustness of the regression results.

Table 2 summarizes baseline characteristics and the analysis of the interaction between waist-to-hip ratio, insulin resistance, and AMH concentrations.

## 4. Discussion

Polycystic ovary syndrome is a heterogeneous disorder with diverse clinical manifestations, necessitating individualized management that accounts for comorbidities such as obesity, metabolic disturbances, and infertility. With the growing recognition of AMH as a potential diagnostic marker in PCOS, clarifying the factors that modulate its levels is of critical importance. Although age, ethnicity, and hormonal contraception are established determinants of AMH, the influence of adipose tissue distribution on AMH remains uncertain. Given the rising prevalence of obesity, especially central obesity, and its frequent coexistence with PCOS, elucidating these relationships is particularly important. Furthermore, population-specific variability in AMH underscores the need for studies in the Eastern European population.

As previously indicated, central obesity frequently occurs in women with PCOS [12]. Multiple studies have shown that central adiposity is more strongly associated with insulin resistance in PCOS than overall body mass index. In this context, the waist-to-height ratio has emerged as a robust and reliable predictor of insulin resistance in women with PCOS, highlighting its clinical utility in identifying patients at higher metabolic risk [13,14,15,16].

The literature emphasizes that central obesity in PCOS is driven by multiple interconnected mechanisms, including insulin resistance, hyperandrogenism, chronic inflammation, oxidative stress, and gut microbiota dysbiosis. Visceral adiposity plays a central role by promoting metabolic and endocrine disturbances, which in turn exacerbate ovarian dysfunction [17]. In women with PCOS, adipose tissue exhibits impaired storage capacity, altered adipogenesis, disrupted insulin signaling and glucose transport, dysregulated lipolysis, and abnormal adipokine/cytokine secretion, all contributing to systemic insulin resistance and chronic low-grade inflammation. Epigenetic mechanisms, including DNA methylation and microRNA regulation, further influence adipose tissue function in PCOS [18].

Building on this evidence, we investigated the association between central adiposity and AMH concentrations, with particular emphasis on the modifying role of insulin resistance. Our analyses revealed a significant interaction between WHR and insulin resistance, indicating that the relationship between central adiposity and AMH differs according to insulin resistance status rather than being the same in all participants. Specifically, in insulin-resistant women, higher WHR was associated with lower AMH concentrations, whereas in women without insulin resistance, higher WHR was associated with higher AMH levels. No significant main effects of WHR or insulin resistance were observed when considered in isolation, underscoring that the association between central adiposity and ovarian reserve is conditional on insulin resistance status.

This observation appears particularly valuable, given that few studies in the literature have attempted to assess the correlation between AMH levels and waist-to-hip ratio (WHR). Nevertheless, these findings are consistent with prior reports emphasizing the negative impact of visceral adiposity on ovarian reserve. Xying Zeng et al., highlighting the uniqueness of their investigation, reported a negative correlation between AMH levels in women with PCOS and central obesity [19]. No association was observed between AMH levels and gynoid obesity. According to these researchers, ovarian reserve, directly reflected by AMH levels, decreases due to the detrimental effect of visceral adipose tissue on follicular function [19].

Xue Li et al. also reported a negative correlation between AMH and WHR; however, unlike our study, they did not consider the moderating role of insulin resistance [20].

In contrast, Aalpona et al. did not observe any association between AMH levels and WHR [21]. Caglar et al. also investigated the relationship between anthropometric parameters, such as WHR, and AMH. However, unlike our study, which focused exclusively on overweight and obese women, their analysis included only women with normal body weight, making direct comparison of the results challenging [22].

The relationship between WHR and AMH was also investigated by Carosso et al., who did not find a significant association between these parameters. However, their study population consisted of a small cohort of women undergoing IVF who did not have PCOS, making it difficult to directly compare their results with ours [23].

Visceral adiposity has been linked to metabolic disturbances that may negatively affect ovarian function [17,18]. Insulin resistance and hyperinsulinemia promote androgen overproduction, impair follicular development, and alter AMH secretion. Additionally, adipose tissue dysfunction in PCOS is characterized by impaired lipid storage, dysregulated adipokine and cytokine secretion, chronic low-grade inflammation, oxidative stress, and epigenetic modifications, all of which may further compromise follicular function and ovarian reserve [24,25]. These mechanisms may provide a biological rationale for the moderating effect of insulin resistance on the WHR–AMH relationship observed in our study group.

In this context, the association between adipose tissue distribution and AMH in PCOS may be mediated by intertwined metabolic, endocrine, and inflammatory mechanisms. Visceral adiposity, a hallmark of the PCOS metabolic phenotype, is closely linked to insulin resistance and compensatory hyperinsulinemia, which enhance ovarian androgen production and contribute to follicular arrest [17,18]. This results in the accumulation of small antral follicles, the primary source of AMH secretion by granulosa cells [3,4]. Moreover, adipose tissue dysfunction in PCOS, characterized by altered adipokine secretion and chronic low-grade inflammation, may directly impair granulosa cell function and modulate AMH expression [24,25]. These disturbances, acting within the ovarian microenvironment, may alter both folliculogenesis and AMH dynamics. Collectively, these mechanisms support the concept that fat distribution, rather than overall adiposity alone, may play a critical role in determining AMH levels in women with PCOS.

The observed interaction between waist-to-hip ratio and insulin resistance in relation to AMH may reflect context-dependent effects of metabolic dysfunction on ovarian follicular dynamics. In insulin-resistant states, hyperinsulinemia may enhance ovarian androgen production and promote follicular arrest, potentially reducing AMH secretion via impaired granulosa cell function [1]. In contrast, in the absence of insulin resistance, central adiposity may be associated with compensatory endocrine adaptations that maintain or increase the pool of small antral follicles, resulting in higher AMH levels [26]. Differences in adipokine secretion and inflammatory activity between metabolic phenotypes may further contribute to the bidirectional pattern observed in our study. Overall, these findings suggest that the relationship between central adiposity and AMH may, at least in part, depend on metabolic phenotype, particularly insulin sensitivity status.

In summary, we observed that insulin resistance may serve as a key moderator of the relationship between central adiposity and ovarian reserve, measured by AMH levels, in women with PCOS. Specifically, elevated WHR was associated with lower AMH only in women with insulin resistance, whereas the opposite pattern was seen in those without insulin resistance. These findings suggest that the combined assessment of insulin resistance and central adiposity may provide additional insight into the interpretation of AMH levels in women with PCOS. However, these observations should be interpreted as exploratory, and further longitudinal studies are required to determine their potential clinical relevance.

## 5. Strengths and Limitations

A strength of this study is that a very good balance was achieved across all covariates, and the criteria for patient selection were clearly defined. Focusing on the Eastern European population ensures the findings are relevant to the studied population and may have potential implications for clinical research. However, given the observational design, the findings should be interpreted as exploratory and hypothesis-generating rather than directly applicable to clinical decision-making. This study has also several limitations, including its observational design and moderate sample size after matching, which limited power to detect small main effects. Although propensity score matching reduced confounding by age and BMI, residual confounding cannot be excluded. Insulin resistance and WHR were analyzed as categorical variables, which may have obscured more subtle dose–response relationships. AMH was assessed cross-sectionally, precluding evaluation of temporal changes. The lack of adjustment for additional phenotypic variables, such as androgen status and ovarian morphology, also represents a limitation of the study. Future studies could address this limitation by incorporating systematically collected androgen measurements and performing analyses in more comprehensively phenotyped cohorts. In addition, to maintain clarity and readability of the tables, insulin and glucose parameters, as well as other components of the full PCOS diagnostic criteria, were not included. Finally, causal inferences cannot be drawn, and the findings may not be generalizable beyond women aged 18–39 years with similar metabolic profiles.

Future research is needed to validate these associations in longitudinal cohorts and to determine whether they have any potential clinical utility. We plan to build on these findings in future studies by further exploring the moderating role of insulin resistance in the relationship between central adiposity and AMH.

## 6. Conclusions

Taken together, our findings demonstrate a significant interaction between waist-to-hip ratio and insulin resistance in relation to AMH concentrations in women with polycystic ovary syndrome. No significant main effects of either WHR or insulin resistance were observed in isolation. Instead, the association between central adiposity and AMH depended on insulin-resistance status: higher WHR was linked to lower AMH in insulin-resistant women, whereas in women without insulin resistance, higher WHR was associated with higher AMH levels.

These results suggest that metabolic status should be considered when interpreting AMH concentrations and assessing ovarian reserve in women with PCOS. In particular, insulin sensitivity may represent an important contextual factor alongside body weight in relation to these associations.

## Figures and Tables

**Table 1:** Baseline characteristics of the age- and BMI-matched cohort stratified by waist-to-hip ratio (WHR).

Parameters	WHR ≤ 0.85 (n = 52)	WHR > 0.85 (n = 52)	SMD
age [years]	23.8 ± 5.6	23.8 ± 5.6	SMD = 0.00
BMI [kg/m^2^]	25.9 ± 4.3	26.0 ± 4.4	SMD = 0.02
AMH [ng/mL]	7.30 ± 3.87	6.65 ± 3.66	-
lnAMH	1.86 ± 0.52	1.76 ± 0.52	-
Insulin resistance n (%)	34 (65.4%)	39 (75.0%)	-

**Table 2:** Interaction between waist-to-hip ratio, insulin resistance, and AMH in PCOS.

Analysis	Comparison/Parameter	Estimate	95% CI	*p*-Value
**Descriptive lnAMH**	WHR ≤ 0.85 (IR+)	1.95 ± SD	-	-
	WHR > 0.85 (IR+)	1.65 ± SD	-	-
	WHR ≤ 0.85 (IR−)	1.71 ± SD	-	-
	WHR > 0.85 (IR−)	2.10 ± SD	-	-
***t*-test (lnAMH)**	WHR > 0.85 vs. ≤ 0.85	Δ = −0.10	[−0.30; 0.10]	0.31
**Two-way ANOVA**	WHR	F(1,100) = 1.13	-	0.29
	Insulin resistance	F(1,100) = 0.46	-	0.50
	**WHR × insulin resistance**	**F(1,100) = 10.81**	-	**0.001**
**Multivariable regression of lnAMH after propensity score matching)**	WHR > 0.85 (IR+)	−0.30	[−0.52; −0.08]	0.008
	Insulin resistance (IR− vs. IR+)	−0.24	[−0.54; 0.05]	0.10
	**WHR × IR** **−**	**+0.69**	**[0.32; 1.06]**	**<0.001**
**Regression (AMH %)**	WHR > 0.85 (IR+)	−26%	[−41%; −8%]	-
	**WHR > 0.85 (IR−)**	**+74%**	**[+38%; +190%]**	-

## Data Availability

The original contributions presented in this study are included in the article. Further inquiries can be directed to the corresponding author.
